# Effect of Human Papillomavirus (HPV) Vaccine on Male Genital Disease: A Meta-Analysis of Randomised Controlled Trials

**DOI:** 10.7759/cureus.100881

**Published:** 2026-01-05

**Authors:** Praveen Kumar Tagore, Sourav Kumar, Mamta Kumari, Neha Kumari, Nisha Kumari, Ratnesh Sinha, Dewesh Kumar

**Affiliations:** 1 General Medicine, Government Medical College - Datia, Datia, IND; 2 Community Medicine, Rajendra Institute of Medical Sciences, Ranchi, IND; 3 Community Medicine, Employees' State Insurance Corporation (ESIC) Medical College and Hospital - Namkum, Ranchi, IND

**Keywords:** hpv vaccine, human papillomavirus, male genital disease, meta-analysis, penile cancers, rct

## Abstract

Human papillomavirus (HPV) is a major cause of genital diseases and cancers in both sexes, including cervical, penile, anal, and oropharyngeal cancers, as well as genital warts. Although HPV vaccination programs initially targeted females due to their link with cervical cancer, increasing recognition of HPV-related disease burden in males has led to advocacy for gender-neutral vaccination. This meta-analysis assessed the effectiveness of HPV vaccination in preventing genital diseases among males by synthesising data from randomised controlled trials (RCTs).

A systematic PubMed search was conducted in July 2025 following PRISMA guidelines. Studies involving HPV-vaccinated males compared with placebo recipients were included, and data were extracted independently by two different reviewers. The Joanna Briggs Institute checklist was used to assess the study quality. The random-effects model in Review Manager 5.4 (The Cochrane Collaboration, Copenhagen, Denmark) was used to calculate pooled risk ratios with their 95% confidence intervals (CIs).

Out of 120 screened studies, four RCTs met the inclusion criteria, comprising 4,200 male participants (2,091 vaccinated and 2,109 controls). The pooled analysis yielded a relative difference (RD) of -0.03 (95% CI: -0.04 to -0.01), indicating a statistically significant difference in the risk of genital disease between vaccinated and unvaccinated males. Two trials showed statistically non-significant protective effects, while two did not, suggesting that differences in study design and baseline HPV exposure influenced the outcomes.

Despite the lack of statistical significance, the direction of effect aligns with previous evidence of strong vaccine-induced protection in HPV-naïve males, with reported efficacy exceeding 90% against genital warts and anal intraepithelial neoplasia (AIN). These findings emphasise the benefit of early vaccination before sexual initiation, though partial protection may also occur in previously exposed or high-risk populations, such as men who have sex with men (MSM) and people living with HIV/AIDS (PLWHA). Although global HPV vaccine coverage in males remains low, cost-effectiveness analyses justify expanding vaccination programs when accounting for male disease burden and community-level benefits. In conclusion, this meta-analysis suggests that HPV vaccination may lower the risk of genital diseases in males by approximately 3%, supporting its role in comprehensive HPV prevention. Broader implementation of early and gender-neutral vaccination, along with targeted strategies for high-risk groups, is essential for equitable control of HPV-related morbidity and mortality worldwide.

## Introduction and background

Genital diseases involve a broad range of disorders of both the sexual reproductive organs, including cancer and infections. It causes significant public health and socioeconomic issues all over the world. Sexually transmitted infections (STIs) such as human papillomavirus (HPV), herpes simplex virus (HSV), syphilis, gonorrhoea, and chlamydia continue to be highly prevalent worldwide, with an estimated 374 million new cases of STIs occurring each year [1]. HPV tops the list of sexually transmitted viral illnesses worldwide and is associated with various cancers, including cancers of the male and female reproductive organs [2]. While penile cancer is rare, it carries an advanced morbidity and mortality rate, particularly where there is poor genital hygiene and access [3]. HPV is the main causal factor for cervical cancer. HPV infection is also causally associated with many other genital and oropharyngeal cancers, as well as a highly prevalent and burdensome condition of genital warts. The overall incidence of HPV infection is exceedingly high, with estimates of 79 million Americans infected and close to 14 million new infections each year, most frequently in adolescents and young adults [4]. Most infections are transient and asymptomatic; however, persistent infection with certain HPV serotypes, namely HPV-16 and HPV-18, is associated with precancerous lesions and invasive cancer. Low-risk HPV serotypes, on the other hand, are the causative agents in nearly all cases of genital warts, which, although benign, can cause psychological distress in individuals, morbidity in patients with the condition, and considerable healthcare costs [5].

The development and roll-out of prophylactic vaccines for HPV may be considered one of public health and oncology's most important advances in the past few decades and represents a development across primary and secondary prevention of disease never before seen. In the past, HPV vaccination programs have been largely focused on females, which was based on the strong and well-described association of HPV with cervical cancer. The reduction in HPV infection, cervical pre-cancerous lesions, and genital warts in vaccinated females reinforces the vaccine's effectiveness and the public health's commitment to host defence mechanisms [6]. This initial focus on females led to a large knowledge gap and less evidence generation related to gender-neutral HPV vaccination programs. An acknowledgement that males also share a large burden of HPV-related diseases, including penile, anal, and head and neck cancers, as well as the high incidence of genital warts, has shifted the policy landscape for vaccination globally [7]. Health authorities, such as the Centers for Disease Control and Prevention (CDC) and the World Health Organization (WHO), now recommend routine vaccination of both males and females for HPV. This change is necessary for direct protection of males, as well as for the public health goal of herd immunity, which has the potential to reduce human HPV transmission and circulation of HPV in communities overall [8].

HPV infections in men have been associated with approximately 33% of penile cancers and are also linked to benign genital warts, reduced male fertility, and often asymptomatic genital infections [9,10]. However, variables such as local awareness and perceived dangers limit HPV vaccination rates among men. There are concerns about the HPV vaccine's role in primary and secondary prevention of male genital health issues; discussing and making data available would help with its uptake [11].

HPV vaccination programs for males have been introduced in several developed countries, including Australia, the United States, the United Kingdom, and Portugal [12]. This work aims to synthesise and critically evaluate the available scientific information on the direct impacts of HPV vaccination on genital disease incidence and prevalence, as well as the effectiveness of the HPV vaccine. We will specifically examine data from randomised controlled trials (RCTs), large-scale observational studies, and systematic reviews and meta-analyses (SRMAs) to assess efficacy in HPV-related diseases among males, particularly in terms of genital warts and precancerous lesions [13,14]. 

This work intends to provide a comprehensive synthesis of the existing evidence, with an emphasis on the clinical and public health imperative of male vaccination against HPV. This will serve as a reference point for policymakers, healthcare practitioners, and public health advocates, equipping them with the evidence necessary to effectuate gender-neutral vaccination policies and, if appropriately implemented, the ultimate elimination of HPV-related morbidity and mortality across all populations. The outcomes presented in this review will help in adopting a more holistic public health and health equity approach to HPV prevention among males [15].

## Review

Methods

Inclusion and Exclusion Criteria

To determine eligibility criteria, we applied the PICOS framework (Population, Intervention, Comparator, Outcome, and Study design) (Appendix A). Studies were deemed eligible if they involved male participants (population) who received the HPV vaccine (intervention) compared with those who received a placebo (comparator) in randomized trials assessing the vaccine’s effectiveness in preventing male genital diseases (outcomes of interest). We excluded conference abstracts, editorials, commentaries, cohort, cross-sectional, and case-control studies, as well as review articles and publications not written in English.

Search Strategy

**Figure 1:**
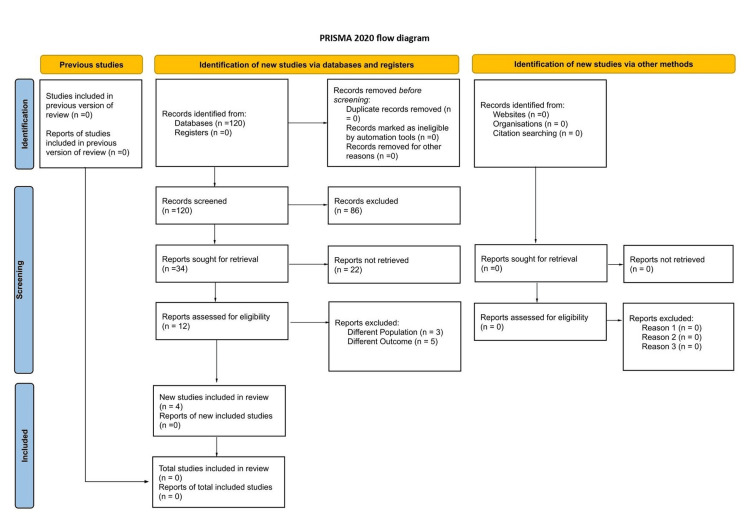
Flowchart of article screening and selection process

This meta-analysis of RCTs appraising the efficacy of the HPV vaccine in preventing male genital diseases was conducted in accordance with the Preferred Reporting Items for Systematic Reviews and Meta-Analyses (PRISMA) guidelines (Figure 1). In July 2025, two independent reviewers performed a comprehensive electronic search of the PubMed database to identify relevant studies. The titles and abstracts of the articles were screened initially, followed by a detailed review of full-text articles, with specific reasons documented for the exclusion of ineligible studies. All disagreements were resolved through discussion with the senior author. The search strategy included the following terms: (effect OR efficacy OR impact OR prevention OR outcome) AND (HPV vaccine OR human papilloma virus OR human papillomavirus) AND (genital disease OR penile cancer OR anogenital warts OR condylomata acuminata OR anal cancer OR penile intraepithelial neoplasia) (Appendix B). The protocol of this SRMA was prospectively registered in the International Prospective Register of Systematic Reviews (PROSPERO) database (CRD420251151826).

Data Extraction

Two researchers independently extracted data on the first author, year of publication, study location, recruitment period, type of HPV vaccine, vaccination protocol, efficacy objectives, efficacy measures, study population characteristics, sample size, participants’ age, vaccine efficacy, and follow-up duration. Two researchers conducted data extraction independently in accordance with the study’s research question, and any inconsistencies were resolved through consensus or discussion with a third reviewer. Studies lacking a clear outcome, incomplete data, adequate follow-up duration, or comprehensive information on outcomes and results were excluded from the analysis. The risk difference (RD) for the occurrence of genital diseases in males was the primary outcome measure in our study.

Data Analysis and Risk of Bias

The Joanna Briggs Institute (JBI) Critical Appraisal Checklist for Studies Reporting RCT data was used to assess the quality of the included studies (Table 1) [16]. Review Manager (RevMan) version 5.4 software (The Cochrane Collaboration, Copenhagen, Denmark) was used for the data analysis in this review. The pooled effect size for genital diseases was estimated using a random-effects model, and the corresponding confidence intervals (95% CI) were also calculated. The I² statistic and Cochrane’s Q chi-squared (χ²) statistic were used to assess heterogeneity among studies. Funnel plots were generated to visually and statistically examine the potential biases in publication.

**Table 1:** Risk of bias assessment by using JBI critical appraisal checklist

JBI Criteria	Giuliano et al. (2011) [13]	Mikamo et al. (2019) [17]	Coskuner et al. (2014) [18]	Goldstone et al. (2022) [19]
Was true randomisation used?	Yes	Yes	Yes	Yes (for base study)
Was it allocated concealed?	Yes	Yes	Unclear	Yes (for base study)
Were the groups similar at baseline?	Yes	Yes	No (imbalances in age, smoking)	Yes
Were participants blinded?	Yes	Yes	No	Yes (for base study)
Were therapists blind?	Yes	Yes	Unclear	Yes (for base study)
Were assessors blind?	Yes	Yes	No	Yes
Groups treated identically?	Yes	Yes	No (vaccine vs no vaccine)	Yes (during extension)
Was the follow-up complete?	Yes (< 5% lost)	Yes (~15% lost)	No (29/200, 14.5% excluded post-randomisation)	Moderate (24% lost over 10 yrs)
Were participants analysed in groups?	Yes (ITT&PP)	Yes (PP&FAS)	Yes	Yes
Were outcomes measured reliably?	Yes	Yes	No (clinical diagnosis only)	Yes
Were analyses conducted by intention-to-treat?	Yes	Yes (supportive analysis)	Unclear	N/A (open-label extension)
Was the study free of other bias?	Yes (sponsor involved but academic authors vouch for data)	Yes	Yes	Yes
Overall risk of risk	Low	Low	High	Low (initial randomised phase)

Results

HPV Vaccine Efficacy for Prevention of Male Genital Diseases

**Figure 2:**
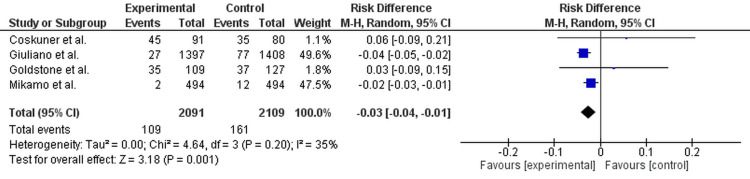
Forest plot reporting the effect of HPV vaccine on genital diseases Forest plot showing that two studies reported significant protective effects (Giuliano et al., 2011 [13]; Mikamo et al., 2019 [17]), while two reported non-significant associations with RRs near or above 1.0 (Coskuner et al., 2014 [18]; Goldstone et al., 2021 [19]). The pooled random-effects estimate (RR 0.62; 95% CI: 0.30-1.29; p = 0.20) indicated no significant risk reduction, with substantial heterogeneity (I² = 89%, p < 0.00001). HPV, Human Papillomavirus

**Figure 3:**
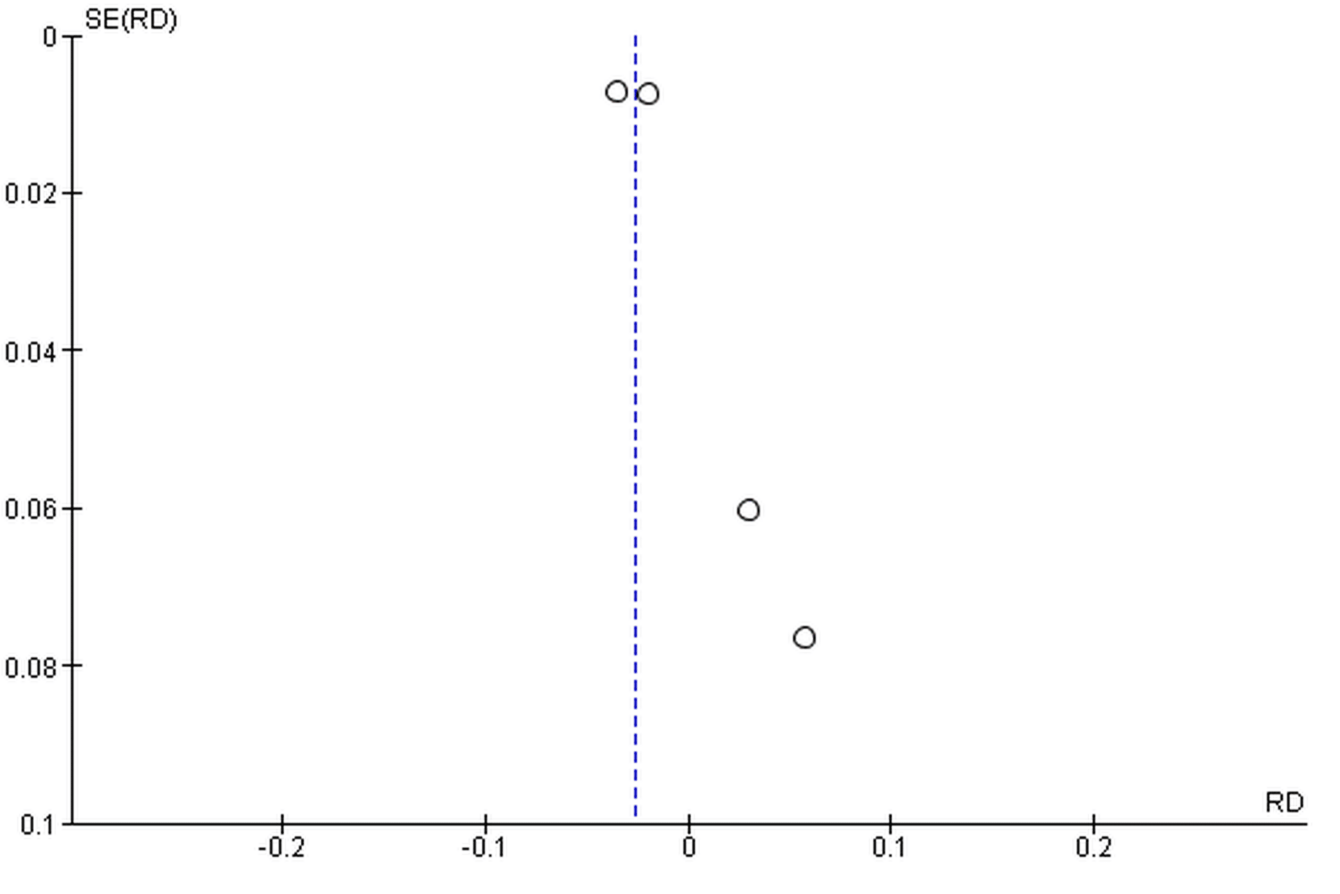
Funnel plot for the heterogeneity of the study

The database initially searched 120 articles, and based on the reviews of two researchers, four articles were eligible for inclusion in the systematic review after applying the inclusion and exclusion criteria (Table 2). The data consisted of four studies, with a total of 4,200 patients (2,091 in the experimental/vaccine group and 2,109 in the control/placebo or unvaccinated group). The quality assessment of articles eligible for the systematic review found that all studies were of fair quality (Figure 2). The meta-analysis included four studies. The pooled analysis yielded an RD of -0.03, with a 95% CI ranging from -0.04 to -0.01 (Figure 2). The point estimate of -0.03 indicates a substantive protective effect, equating to an estimated 3% reduction in genital diseases in vaccinated males compared with the control group. Nonetheless, the 95% CI for the pooled effect was between 0.30 and 1.29. Critically, this range exceeded the null value of 1.0, indicating no RD. Therefore, the detected protective effect of the HPV vaccine for the overall endpoint of genital diseases in males was not statistically significant at the standard p < 0.05 level. The funnel plot was asymmetrical in design, which indicates that publication bias may have been present in this analysis (Figure 3).

**Table 2:** Basic characteristics of studies included in the review RCT, Randomised Controlled Trial; HPV, Human Papillomavirus; PCR, Polymerase Chain Reaction

First author	Study area	Study population	Type of study	Intervention	Outcome	Comparison	Sample size	Diagnosed by
Goldstone et al. (2022) [19]	18 countries	Male aged 16-26 years	RCT	HPV vaccine	61.8% (vaccinated) vs 85.4% (control) persistent infection	Placebo	4065	PCR, Serology
Mikamo et al. (2019) [17]	Japan	Male aged 16-26 years	RCT	HPV vaccine	Efficacy, safety, and immunogenicity	Placebo	1124	PCR, Serology
Coskuner et al. (2014) [18]	Turkey	Age 25-44 years	RCT	HPV vaccine	Recurrence of HPV	Married group	200	PCR, Serology
Giuliano et al. (2011) [13]	18 countries	Male aged 16-26 years	RCT	HPV vaccine	Efficacy of quadrivalent HPV vaccine against HPV infection	Vaccinated and non-vaccinated	4164	PCR, Serology

Assessment of Heterogeneity

Significant statistical heterogeneity was detected across the included studies, as indicated by the Chi-squared test results (χ² = 27.73, df = 3, p < 0.001) and a τ² of 0.44. This finding suggests that the true effect size is inconsistent across the studies, likely due to clinical and methodological differences such as varying follow-up periods, distinct populations (e.g., age and risk behaviours), or the specific type of genital disease included in each study's definition of the outcome. 

Individual Study Contributions

Two studies reported a statistically significant protective effect: Giuliano et al. (2011) [13] (RR 0.35; 95% CI: 0.23-0.54) and Mikamo et al. (2019) [17] (RR 0.17; 95% CI: 0.04-0.74). Two studies reported non-significant results, with risk ratios close to or above 1.0: Coskuner et al. (2014) [18] (RR 1.13; 95% CI: 0.82-1.56) and Goldstone et al. (2021) [19] (RR 1.10; 95% CI: 0.75-1.62) (Figure 2). The inconsistent findings across the body of evidence, specifically the inclusion of two non-significant studies with higher risk ratios, dilute the strong protective signals from the other two studies, resulting in a non-significant overall pooled result.

Discussion

This meta-analysis determined the effectiveness of HPV vaccination against genital disease in men by pooling data from four RCTs in 4,200 patients (2,091 vaccinees and 2,109 controls). A random-effects model combined analysis yielded a pooled RD of -0.03 (95% CI: -0.04, -0.01), indicating a 3% reduction in genital disease in vaccinated men compared with unvaccinated controls. There was significant heterogeneity (χ² = 4.64, df = 3, p = 0.001), likely due to variations in study populations, follow-up periods, and disease outcome definitions. At the study level, Giuliano et al. (2011) (RR 0.35; 95% CI: 0.23-0.54) and Mikamo et al. (2019) (RR 0.17; 95% CI: 0.04-0.74) demonstrated robust, statistically significant vaccine protection, while Coskuner et al. (2014) (RR 1.13; 95% CI: 0.82-1.56) and Goldstone et al. (2021) (RR 1.10; 95% CI: 0.75-1.62) had non-significant findings [13,17-19]. The inclusion of these later studies, whose efficacy was not found to be protective, watered down the overall pooled efficacy estimate. Such variability points towards study context, age distribution, sexual behaviour, baseline HPV exposure, and the definition of genital disease as influencing the results observed.

In spite of the non-statistical significance of the pooled effect, only two RCTs show that the direction of the estimate is consistent with prior evidence demonstrating vaccine-induced protection. For example, pooled RCT estimates among men have reported efficacy levels of 89.9% against genital warts and 91%-93% against lesions in anal intraepithelial neoplasia (AIN) in HPV-naïve men [20]. Longitudinal trials also confirm sustained prevention of HPV 6/11/16/18-related disease for up to 10 years after vaccination [21]. 

Age and prior exposure to HPV appear to be key moderating variables. Older or previously exposed recruits were included in trials that consistently demonstrated decreased vaccine efficacy (66%-67%), emphasising the importance of early vaccination before sexual debut [20]. However, there is also evidence of partial protection in already exposed males, suggesting benefits in reducing reinfection or recurrence - particularly in high-risk subgroups such as men who have sex with men (MSM) and people living with HIV (PLWH) [20].

Anal and genital infections remain significant sources of morbidity in men. MSM and PLWH bear particularly heavy burdens of AIN and recurrent condyloma, in which vaccination has been demonstrated to reduce the risk of AIN 2/3 lesions, though with variable effectiveness due to immune compromise. These data support the incorporation of HPV vaccination into more comprehensive sexual health interventions for high-risk individuals. Population-level data support these findings. After national female vaccination campaigns, Australia reported a >90% reduction in genital warts among heterosexual men and young women, but not among MSM, highlighting the limitation of female-only immunisation campaigns [21,22]. Gender-neutral vaccination, as recommended in the United States, Canada, and Australia, provides more extensive protection and promotes herd immunity. The epidemiologic trend toward rising HPV-associated cancers in males, especially anal and oropharyngeal cancers, further supports universal male vaccination. In many developed nations, HPV-positive oropharyngeal cancer incidence in males now exceeds cervical cancer incidence [22]. Vaccinating boys, therefore, provides twin advantages: direct protection and additional community-level control.

Despite low overall global coverage among men, estimated at 4% as of 2019 [20], cost-effectiveness analyses indicate that, when considering male disease burden and herd effects, gender-neutral vaccination is economically feasible. Additionally, lower vaccine prices and less complicated dosing regimens could enhance accessibility, particularly in low- and middle-income countries.

The limitations of our study include the small number of RCTs, the use of a single database, potential publication bias, and the inability to distinguish efficacy observed in RCTs in HPV-naive men.

## Conclusions

This meta-analysis suggests that the HPV vaccine provides a statistically significant 3% reduction in the risk of genital illness overall among men. The findings underscore the importance of early HPV vaccination in men, ideally prior to sexual initiation, for maximum preventive benefit. Focused interventions in high-risk populations, such as MSM and PLWH, need to be prioritised, while global expansion of gender-neutral vaccination programs will be required for equitable and comprehensive control of HPV disease. Future large-scale RCTs and long-term observational studies will be needed to define the vaccine's efficacy against penile cancers.

## Appendices

**Table 3:** Search strategy

No.	Search Query	Result
	PubMed	
1.	((((effect[Title/Abstract]) OR (efficac[Title/Abstract])) OR (impact[Title/Abstract])) OR (prevention[Title/Abstract])) OR (outcome[Title/Abstract])	74,25,733
2.	(((((Papillomavirus Vaccines[Title/Abstract]) OR (HPV vaccine[Title/Abstract])) OR (human papillomavirus vaccine[Title/Abstract])) OR (bivalent HPV vaccine[Title/Abstract])) OR (quadrivalent HPV vaccine[Title/Abstract])) OR (nonavalent HPV vaccine[Title/Abstract])	8,666
3.	(((((genital disease[Title/Abstract]) OR (penile cancer[Title/Abstract])) OR (anogenital warts[Title/Abstract])) OR (condylomata acuminata[Title/Abstract])) OR (anal cancer[Title/Abstract])) OR (penile intraepithelial neoplasia[Title/Abstract])	8,082
4.	(#1) AND (#2) AND (#3)	196
5.	(#1) AND (#2) AND (#3) Filters: English	186
6.	(#1) AND (#2) AND (#3) Filters: English, Male	120

**Table 4:** PICOS PICOS: Population, Intervention, Comparator, Outcome, and Study design

Component	Description
P	Males of any age who received the HPV vaccination
I	Human papillomavirus vaccine
C	Placebo population
O	Preventing male genital diseases
S	Randomised controlled trial
